# Single-Cell Fluidic Force Spectroscopy Reveals Dynamic Mechanical Fingerprints of Malignancy in Breast Cancer

**DOI:** 10.1021/acsami.4c06335

**Published:** 2024-08-06

**Authors:** Zeina Habli, Ahmad Zantout, Nadine Al-Haj, Raya Saab, Marwan El-Sabban, Massoud L. Khraiche

**Affiliations:** † Neural Engineering and Nanobiosensors Group, Biomedical Engineering Program, Maroun Semaan Faculty of Engineering and Architecture, American University of Beirut, Beirut 1107 2020, Lebanon; ‡ Department of Anatomy, Cell Biology, and Physiological Sciences, Faculty of Medicine, American University of Beirut, Beirut 1107 2020, Lebanon; § Department of Pediatrics, Stanford University School of Medicine, Palo Alto, California 94304, United States

**Keywords:** single-cell force spectroscopy, cell adhesion, metastasis, breast cancer, cancer mechanics

## Abstract

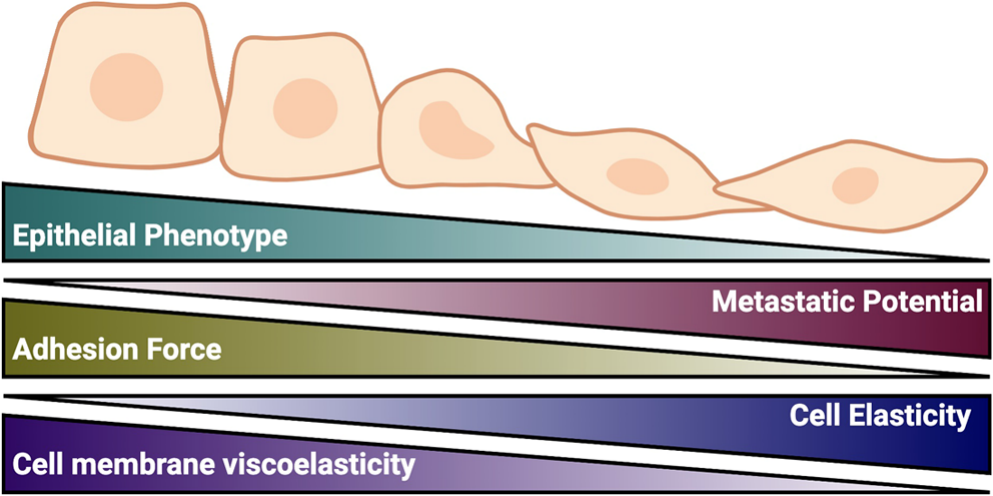

The interplay between cancer cell physical characteristics and metastatic potential highlights the significance of cancer cell mechanobiology. Using fluidic-based single-cell force spectroscopy (SCFS), quartz crystal microbalance with dissipation (QCM-D), and a model of cells with a spectrum of metastatic potential, we track the progression of biomechanics across the metastatic states by measuring cell–substrate and cell-to-cell adhesion forces, cell spring constant, cell height, and cell viscoelasticity. Compared to highly metastatic cells, cells in the lower spectrum of metastatic ability are found to be systematically stiffer, less viscoelastic, and larger. These mechanical transformations in cells within a cluster correlate with cells’ metastatic potential but are significantly absent in single cells. Additionally, the response to chemotherapy is found to be highly dependent on cell viscoelastic properties in terms of both response time and magnitude. Shifts in cell softness and elasticity might serve as mechanoadaptive mechanisms during cancer cell metastasis, contributing to our understanding of metastasis and the effectiveness of potential therapeutic interventions.

## Introduction

Solid tumors are often detected due to their increased stiffness and rigidity compared to normal tissues (i.e., detection by palpation); however, the mechanical properties of tumors can become more complex as they progress.^1−3^ During cancer invasion and metastasis, the attributes of cancer cells are altered, dictating changes in their biomechanical properties and influencing their proliferation, differentiation, migration, contractility, and apoptosis. The deviations from mechanical homeostasis are associated with pronounced cell invasiveness and are coupled with the reorganization of the extracellular matrix (ECM), reduced cell–cell and cell–matrix adhesion, increased cell elasticity and deformability, and improved cell motility.^4−6^ These alterations have been related to differences in the cytoskeletal structure and membrane viscosity, which affect the level of cell softness and the degree of cell malignancy and metastatic states.^3,7−10^ This malignant transformation and mechanical remodeling are accompanied by epithelial-to-mesenchymal transition (EMT) and happen under the influence of upregulation, downregulation, or even inhibition of genes and tumor marker expressions such as integrins, actin, fibronectin, Rho GTPase, gap junction proteins, and EMT markers, among others.^11^

Gap junction intercellular communication (GJIC) is assembled from connexin (Cx) proteins, which are transmembrane proteins involved in cell–cell communication and the exchange of ions and metabolites between adjacent cells.^12^ Connexin 43 (Cx43), a building block of the channel-forming gap junctions (GJs), exhibits spatiotemporal expression patterns and plays an essential role in mammary tissue development, differentiation, and tumor suppression.^13^ Impaired Cx43 expression and localization may alter the function of the mammary gland and lead to cancer onset and progression, where tumor cells physically detach from their tumor microenvironment, invade tissues and ultimately colonize distant organ sites.^14,15^ On the other hand, when Cx43 expression is restored, it mediates active GJIC between adjacent cells and aids tumor cells in their interaction with the endothelium, thereby enhancing their intravasation/extravasation.^16,17^ We recently reported that Cx43 upregulation in metastatic human breast epithelial MDA-MB-231 cancer cells resulted in increased expression of epithelial markers like E-cadherin and ZO-1, and the sequestration of β-catenin at the cell membrane *in vitro*, in addition to the attenuation of primary tumor growth and malignancy potential of these cells *in vivo*. On the other hand, Cx43 silencing rendered these breast cancer cells with a mesenchymal-like morphology with increased N-cadherin expression *in vitro* and a more aggressive metastatic phenotype *in vivo*.^18,19^

Recent efforts in breast cancer therapy exploring inhibitory pathways involved in modulating cell biomechanical properties like stiffness, elasticity, and adhesivity have recently gained immense attention.^20,21^ Docetaxel (DTX), a microtubule-stabilizing agent, has been shown to disrupt intercellular adhesive forces, impairing cytoskeletal adhesion proteins such as actin and integrins, while also altering cellular morphology, division, and motility.^22^ Characterizing the biomechanical properties of cancer cells can provide better insights into the mechanics of tumor onset and metastasis, serve as biomarkers for early cancer detection, and offer new basis for developing new therapies that can modulate cell mechanical cues for enhanced treatment.

In this study, we investigated the correlation between the biomechanical properties of MDA-MB-231 breast epithelial cancer and their metastatic potential using single-cell force spectroscopy (SCFS). Our findings revealed that the downregulation of Cx43 led to the softening of cancer cells, which was associated with diminished cell–cell adhesion, increased elasticity and deformability, and a heightened potential for malignancy and aggressiveness. On the other hand, upregulating Cx43 was associated with tight cell–cell adhesion, stiffness, and rigidity, with reduced malignant potential. Finally, the time course of biomechanical changes in response to DTX indicated increased cellular stiffness in aggressive and more malignant cell subtypes.

## Results

### Regulation of Cx43 Expression Induces Morphological Changes Correlated with Metastatic Potential

**Figure 1:**
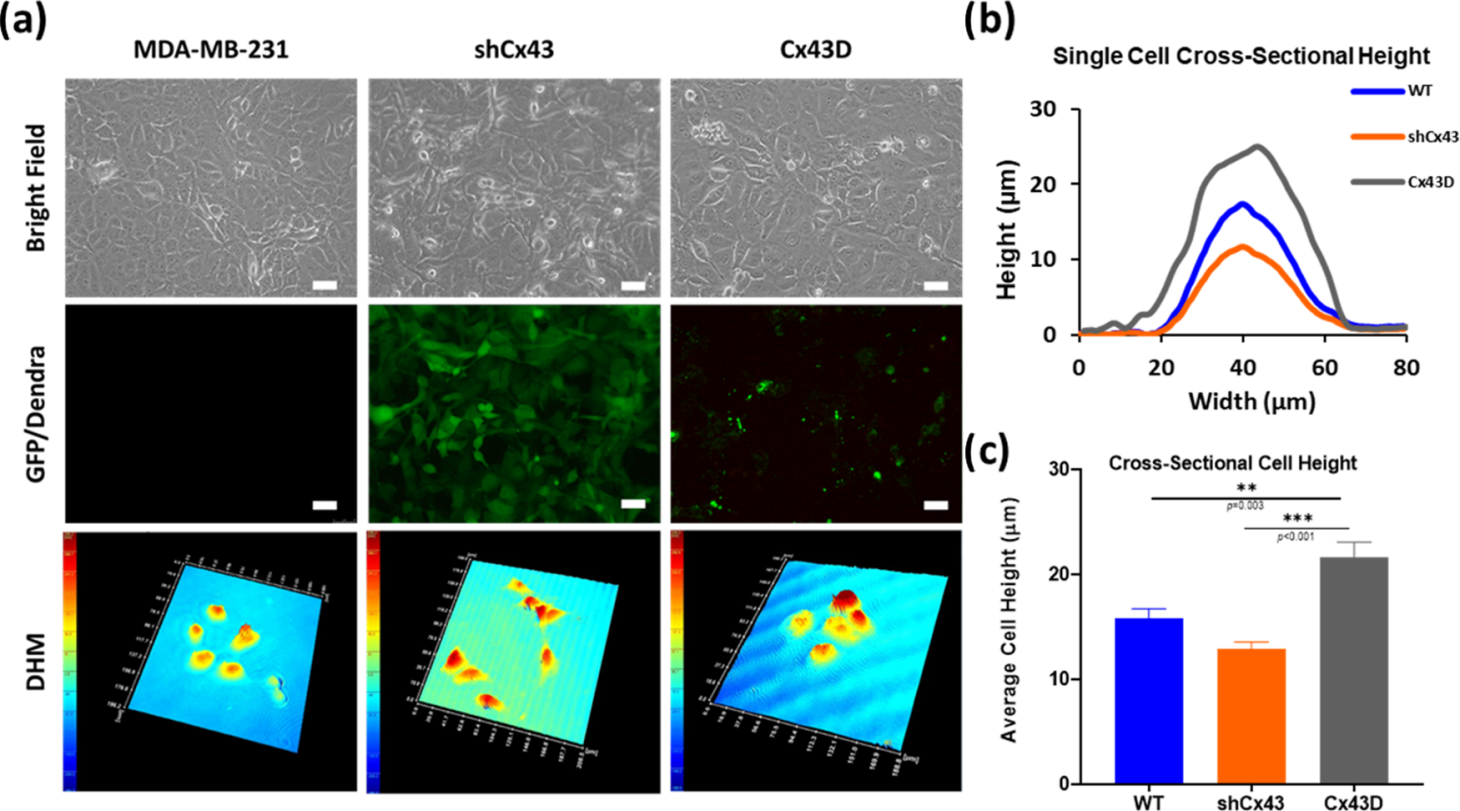
Cx43 gene regulation in MDA-MB-231 cells affects cell size and morphology. (a) Representative images of parental MDA-MB-231 (WT), shCx43, and Cx43D cells. Cell morphology and size are shown in the bright microscopy panel. The quality of transfected and transduced MDA-MB-231 cells with shCx43/Cx43D vectors, respectively, is represented in the GFP/Dendra panel. Scale bar: 50 μm. The bottom panel represents three-dimensional (3D) reconstructed holographic images of the different cell subtypes. (b) Cross-sectional height cell measurements of MDA-MB-231 WT, shCx43, and Cx43D cells, as depicted by the DHM. (c) Average cross-sectional cell height from at least 30 single cells in each Cx43 expression condition. The values depicted are the mean ± standard error of the mean (SEM) from three separate experiments, and at least 30 cells were evaluated per condition. *** denotes a *p*-value <0.001, ** denotes a *p*-value <0.01, and * denotes a *p*-value <0.05 compared to different conditions using analysis of variance (ANOVA) followed by the post hoc Tukey honestly significant difference (HSD) test.

The regulation of Cx43 expression induced significant morphological changes in MDA-MB-231 cells where shCx43 cells maintained a mesenchymal-like phenotype, while Cx43D cells acquired more of an epithelial phenotype (Figure 1a, upper and middle panels). We quantitavely assessed this change by measuring the cross-sectional cell diameter and cell height of individual cells in the middle using digital holographic microscopy (Figure 1a, DHM panel). All three cell lines showed almost equal cell diameter size of around 40 μm in all measured cells (Figure 1b) but different average cross-sectional cell heights, with Cx43D cells exhibiting the greatest height (21.61 ± 1.44 μm), followed by WT cells (15.82 ± 0.9 μm) and shCx43 cells (12.9 ± 0.62 μm) (Figure 1c). Note that we have previously established and characterized these three subsets of MDA-MB-231 cells with silenced or upregulated Cx43 expression, recapitulating a triple-negative breast cancer *in vitro* model of the same cell line lineage with varying metastatic potential.^18,19^ The expression of Cx43 was routinely assessed with qPCR in sorted cells and compared to the expression of Cx43 in MDA-MB-231 cells and WT cells (control), as depicted in Figure S1.

### Cellular Metastatic Potential Alters Cell Adhesion, Regulates Cell–Cell Interactions, and Modifies Cellular Elasticity

**Figure 2:**
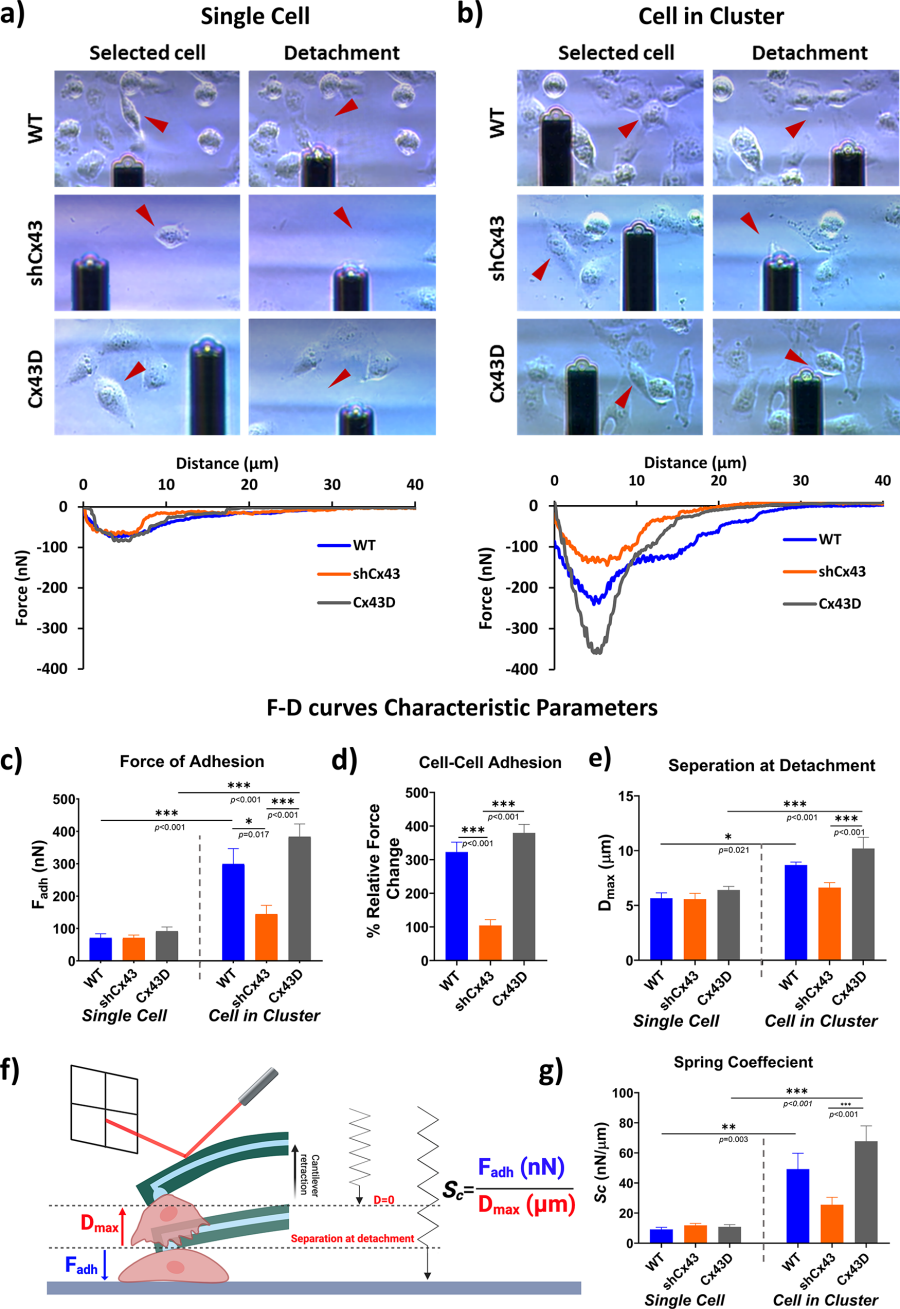
Metastatic state of MDA-MB-231 cells dictates cellular adhesion strength revealed by fluidic-based SCFS. (a) Individual cell, and (b) cell in a cluster (cell adherent to at least two cells) detachment of MDA-MB-231 WT, shCx43, and Cx43D cells. Adhesion forces of single cells reflect cell–substrate adhesion, while those of a cell in a cluster reflect cell–cell adhesion. (c–g) Comparison of *F*–*D* curves characteristic parameters (*F*_adh_, *D*_max_, and *F*_adh_/*D*_max_ ratio, referred to as spring coefficient (*S*_c_)) between MDA-MB-231 cells with varying expression of Cx43 and different cell culture categories (single cells *vs* cells in a cluster). (c) Average adhesion force measured in MDA-MB-231 cells with varying Cx43 expression in single and cluster cell states. (d) Intercellular adhesion forces of MDA-MB-231 cells with varying Cx43 expression are represented by the relative force change between cells in cluster and single-cell states. (e) Average longest cantilever elongation before detachment (represented by *D*_max_) in single and cluster cell states. (f) Spring coefficient (*S*_c_) concept and an illustrative schematic (not to scale) showing the elastic deformation capability of the cell upon pulling it from the surface using a hollow FluidFM cantilever. The ratio of *F*_adh_ (maximum force) over *D*_max_ (longest elongation before detachment) when pulling the cell characterizes cell elasticity potential and can be interpreted from the *F*–*D* curves. Adapted with permission from ref (24) reference # 1488717-1. Copyright 2024 RSC. (g) Average cell elasticity represented by *F*_adh_/*D*_max_ between MDA-MB-231 cells in different culture states. The concept of cell elasticity was adapted from ref (25). The values depicted are the mean ± SEM from three separate experiments, and at least 15 cells were evaluated per condition. *** denotes a *p*-value <0.001, ** denotes a *p*-value <0.01, and * denotes a *p*-value <0.05 compared to different conditions using ANOVA followed by the post hoc Tukey HSD test.

Fluidic force microscopy was utilized to measure the mechanical and biophysical properties of individual cells grown as single cells (not in direct contact with other cells) and on cells within a cluster (fully surrounded by adjacent cells) under similar conditions and confluency. This SCFS setup was used to discern and quantify the impact of the cell–cell interaction, under the influence of Cx43 expression, on the overall adhesion forces in cells on a scale of metastatic potential. Bright-field images of SCFS-targeted cells before and after detachment with corresponding representative force–distance (*F*–*D*) curves registered during the detachment of cells are depicted in Figure 2a,2b and Videos S1–S6. The obtained *F*–*D* curves show substantial progression from a single-cell state to cells in a cluster, signifying the impact of Cx43 on the overall binding. MDA-MB-231 cells in a single-cell state, regardless of Cx43 expression, exhibited the same adhesion strength with an average adhesion force of around 70 nN (Figure 2a, 2c). Surprisingly, MDA-MB-231 cell adhesion in a cluster varied significantly between the different subsets and compared to the single-cell state within the same subset, revealing a notable difference in adhesion between the studied cells and a relevant role of Cx43 in cell–cell adhesion. Cx43D cells showed a significant surge of *F*_adh_ surpassing 380 nN; a similar trend, but to a lesser extent, was observed with the WT cells that had an average *F*_adh_ of 300 nN, both of which are at least four times significantly higher than *F*_adh_ of individual cells that registered 70 nN. Conversely, the highly metastatic shCx43 cells in clusters exhibited only a 2-fold increase in *F*_adh_ compared to a single-cell state averaging around 145 nN (Figure 2b, 2c). While the force measured on single cells implies the adhesion force between the cell and the underlying substrate (*F*_cell-substrate_), the force measured of a cell within a cluster also includes the intercellular adhesion forces (*F*_cell-substrate_ + *F*_cell–cell_). To examine this aspect, intercellular adhesion forces represented as the relative force change between cells in a cluster and cells in a single-cell state can be calculated according to the relative force equation.^23^ The intercellular forces were 230 nN in the control WT cells, 74 nN for the highly metastatic shCx43 cells, and 290 nN for the nonmetastatic Cx43D cells. Moving from an individual cell state to a cell within a group of cells, WT cells showed a 320% increase in force change, shCx43 cells showed a 100% increase in force change, while Cx43D cells showed a ≅400% increase in force change (Figure 2d).

The distance traveled by the cantilever right before the last detachment event gives an implication of the cell elongation capacity until the maximum detachment force (*i.e.*, *F*_adh_) is reached. Individual MDA-MB-231 cells, regardless of Cx43 expression, had similar ranges of *D*_max_ (≅5.5 μm), contrary to cells within a cluster, which varied significantly between the different cell subtypes. The lowest *D*_max_ values were recorded in WT cells and shCx43 cells, averaging around 6.6 and 8.8 μm, which are significantly smaller than the those recorded for Cx43D cells, averaging around 10.2 μm (Figure 2e). As a matter of fact, cells that require a higher *D*_max_ to fully detach usually exhibit a larger adhesion strength, which is consistent with the recorded *F*_adh_ data.

To estimate the stiffness of the cells in this model, the spring coefficient (*S*_c_) derived from *F*_adh_/*D*_max_ is used to estimate the elastic deformation capacity of the studied cells (Figure 2f).^25^ The mean *S*_c_ values of single MDA-MB-231 cells ranged between 9.2 and 11.9 nN/μm with no statistical significance between the different subsets. However, when in clusters, the mean *S*_c_ significantly increased when Cx43 is overexpressed compared to other subsets and compared to single-cell states while showing no significant change when Cx43 expression is downregulated (Figure 2g).

### Cellular Metastatic Potential Controls Cell Viscoelasticity

**Figure 3:**
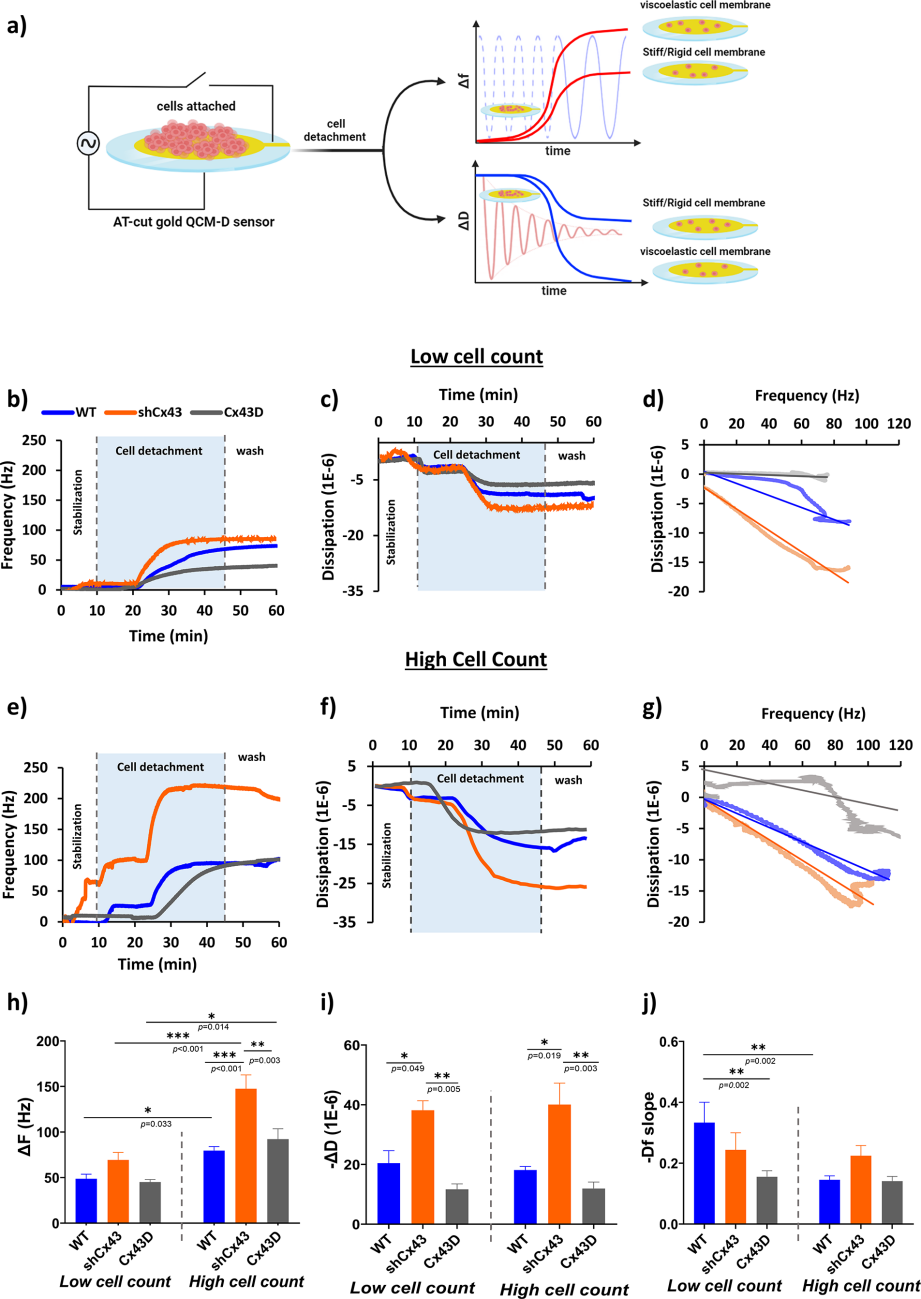
Metastatic state of MDA-MB-231 cells influences cellular viscoelasticity revealed by QCM-D. (a) Illustration of QCM-D cell-based experiments in liquid conditions and the corresponding plots reporting changes in frequency (Δ*F*) and dissipation energy (Δ*D*) *vs* time according to the rigidity or softness of the cell layer. QCM-D utilizes a converse piezoelectric effect: the AT-cut quartz crystal oscillates when an electric voltage is applied. The mechanical oscillations can be transferred into an equivalent electric circuit model, which allows the depiction of a complete description of the oscillation in the presence of a mass and viscous loading. In the case of soft and viscoelastic cell monolayers, the amplitude of the crystal oscillation decreases rapidly due to the high energy losses compared to rigid cell monolayers. (b–j) Real-time QCM-D measurements. (b) Δ*F*- and (c) Δ*D*-responses, and the corresponding (d) *Df* -plots with linear regression of low cell count MDA-MB-231 cells with varying expression of Cx43 undergoing cell detachment. (e) Δ*F*- and (f) Δ*D*-responses and the corresponding (g) *Df*-plots with linear regression of high cell count MDA-MB-231 cells with varying expression of Cx43 undergoing cell detachment. The cells were seeded on top of AT-cut gold-coated quartz sensors for 24 h, after which they were mounted on the modules and washed with PBS for 10 min at a 10 μL/min flow rate. The cells were detached with trypsin at 30 μL/min, and the frequency and dissipation were recorded over a period of 35 min. The cells were then washed with PBS to remove the unbound cells. Change in (h) frequency and (i) dissipation amplitudes and (j) *Df*-slopes for MDA-MB-231 cells with varying Cx43 expression at different cell count states. The values depicted are the mean ± SEM from at least three separate experiments evaluated per condition. *** denotes a *p*-value <0.001, ** denotes a *p*-value <0.01, and * denotes a *p*-value <0.05 compared to different conditions using ANOVA followed by the post hoc Tukey HSD test. The data shown are from the same control and experimental pair.

To better correlate cellular elasticity and viscosity to their metastatic potential and attain a timeline of potential changes in response to treatments, we used a quartz crystal microbalance with dissipation (QCM-D). A representative schematic of time-dependent cell detachment monitored by QCM-D is depicted in Figure 3a, with the corresponding shifts in frequency and dissipation implicated by the viscoelastic state of the cells under study. Upon inspecting cell detachment dynamics with trypsin using QCM-D, we noticed a single-phase QCM-D signal for all cell subtypes with a steep increase in Δ*F* and a sharp decrease in Δ*D*, after which a plateau was reached at 20 min, and remained almost stable even after 1× phosphate-buffered saline (PBS) injection to ensure washing of loosely bound cells as shown in Figure 3b, 3c,3e,3f. Figure 3d,3g shows the *Df* -plots (indicated by the Δ*F*/Δ*D* ratio) of the three MDA-MB-231 subtypes with their respective linearization. The obtained plots are in line with typical frequency and dissipation responses obtained upon cell rounding followed by cell detachment. The recorded frequency and dissipation signals and the derived *Df*-plot behaved almost similarly in the three subtypes and cell densities but with differences in magnitudes.

In low cell count, the rise in Δ*F* was almost similar in all subtypes contrary to high cell count where recorded Δ*F* varied significantly between the subtypes, with shCx43 experiencing the greatest shift in frequency at around 150 Hz, and Cx43 and WT cells demonstrating similar shifts ranging between 80 and 90 Hz (Figure 3h). When comparing Δ*F* between cell-count densities, shCx43 and WT cells demonstrated a significant increase in frequency, with no reported significance in Cx43D cells. On the other hand, in both low and high cell counts, Δ*D* varied significantly among the three subsets, corroborating a unique viscoelastic signature of each cell type (Figure 3i). The highly metastatic shCx43 cells exhibited the highest Δ*D*-response recording around 40 × 10^–6^, while nonmetastatic Cx43D cells registered three times smaller Δ*D*-response averaging around 12 × 10^–6^ and WT cells had an intermediate Δ*D*-response averaging around 20 × 10^–6^. Upon inspecting the *Df*-plots’ slopes, in the low cell count state, shCx43 cells had the smallest slope, averaging at −0.252, followed by WT and Cx43D, having intermediate mean slopes averaging around −0.406. Concurrently, in high cell count experiments, the slopes of *Df*-plots were similar in all three subtypes, with relatively small values ranging between −0.145 and −0.236 (Figure 3j). Interestingly, the slopes varied between the different cell confluencies, where Cx43D and WT had a greater slope in their low cell count states, while shCx43 barely registered any changes between the two cell densities.

### Cellular Metastatic Potential Influences Chemotherapy Sensitivity

**Figure 4:**
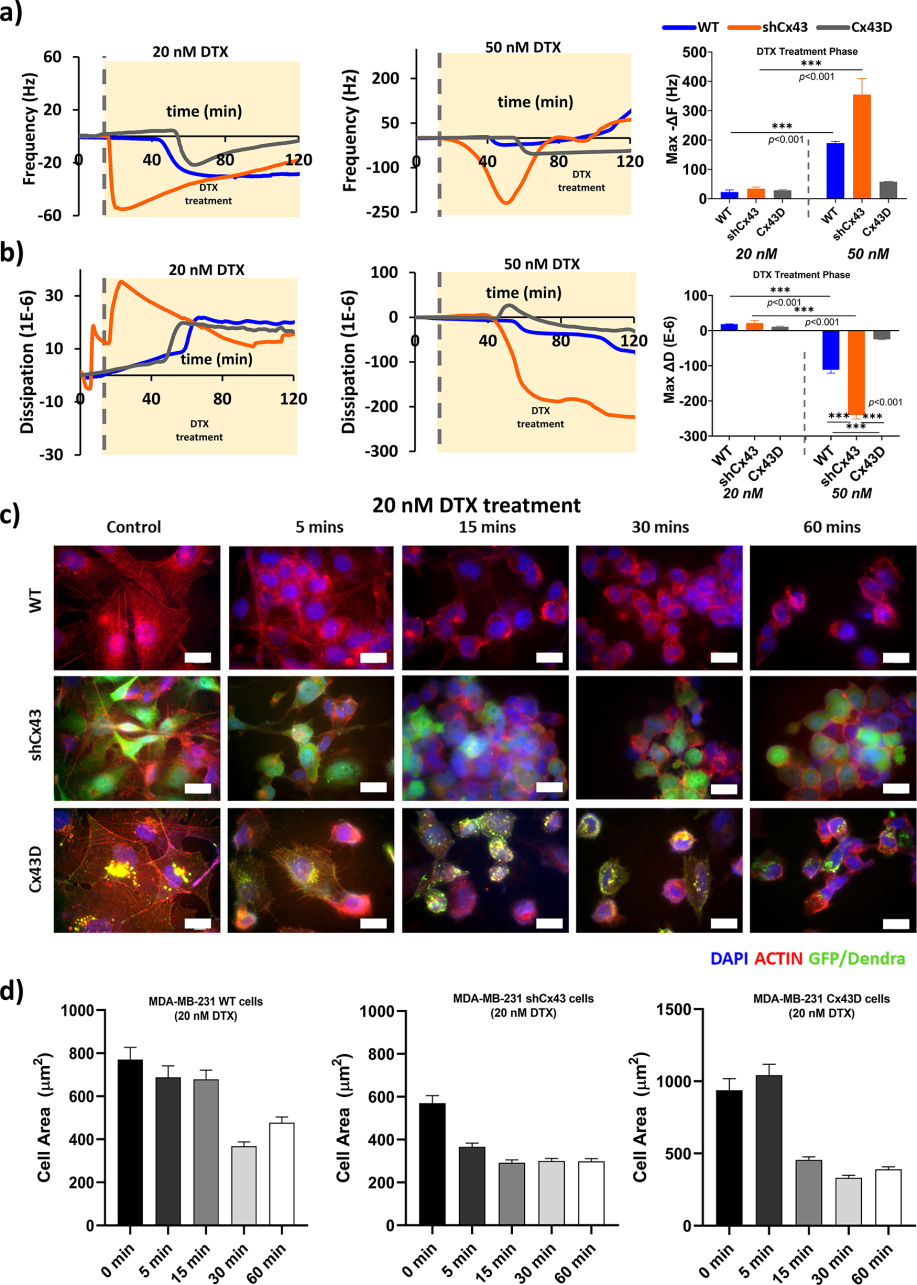
Metastatic state of MDA-MB-231 cells affects cellular real-time responses to DTX treatment. (a) Δ*F*-responses and change in frequency of MDA-MB-231 cells with varying expression of Cx43 to DTX (20 and 50 nM) (b) Δ*D*-responses and change in dissipation of MDA-MB-231 cells with varying expression of Cx43 to DTX (20 and 50 nM). The values depicted are the mean ± SEM from at least three separate experiments evaluated per condition. (c) Confocal images of treated cells at different time points. The cells were stained with actin phalloidin (red), and the nuclei were counterstained with 4′,6-diamidino-2-phenylindole (DAPI) (blue). Scale bar: 20 μm. (d) Cell area (μm^2^) progression upon treatment with DTX at different time points.

Contrary to cell detachment with trypsin, treating the cells with DTX at two different doses (20 and 50 nM) resulted in a two-phase QCM-D response with a steep decrease followed by a gradual increase in Δ*F*, and two vastly different Δ*D*-responses between the doses, with different magnitudes and speed in responses (Figure 4a, 4b). The highly metastatic shCx43 cells demonstrated a rapid, negative, and steep Δ*F*-response only a few minutes into the treatment, contrary to the nonmetastatic Cx43D cells and WT parental cells that showed responses 30 min into the treatment. In fact, shCx43 cells showed the highest negative Δ*F* shift upon treatment with 20 and 50 nM of DTX averaging around 35 and 355 Hz, respectively, after which the frequency went up back to zero, with a much faster response in the 50 nM dose. Conversely, WT and Cx43D cells exhibited the same negative Δ*F* shift responses when treated with DTX: a gradual decrease in Δ*F* followed by a slow increase to the zero offset, with WT exhibiting higher frequency responses with the 50 nM treatment dose. Under similar treatment concentrations, no significance was recorded among the three cell subtypes, but when comparing the two treatment schemes, WT and shCx43 cells showed a significant difference in Δ*F*-response. Notably, the decrease in Δ*F* below the equilibrated levels, contributing to a negative Δ*F*, may indicate cellular stiffening.

Unlike frequency responses, dissipation responses during DTX treatments indicate cell membrane viscoelastic dynamics. When treated with 20 nM DTX, a positive Δ*D*-response of the three MDA-MB-231 subtypes was recorded, indicating the possibility of cellular stiffening due to the chemotherapy treatment. Similar to Δ*F* shift, the highly metastatic shCx43 cells exhibited a rapid sharp increase in Δ*D* contrary to WT and Cx43D subtypes that plateaued at zero for 30 min into treatment and then started to show a gradual increase until reaching stable values. shCx43 cells exhibited the greatest Δ*D*-response averaging around 21 × 10^–6^, followed by WT and then Cx43D with Δ*D*-response averaging around 18 × 10^–6^ and 11 × 10^–6^, respectively. In contrast, treatment with 50 nM DTX resulted in negative Δ*D*-responses with much higher magnitudes opposite to 20 nM DTX treatment. As expected, shCx43 cells exhibited the largest Δ*D*-response averaging around −240 × 10^–6^, a 6-fold difference compared to WT cells and Cx43D cells. Remarkably, Cx43D cells showed a slight increase in dissipation followed by an almost equal decrease with an average of 25 × 10^–6^ in response to DTX treatment. Within the same treatment group, no significance was recorded, but upon comparison of WT and shCx43 cells at the two treatment doses, the recorded shifts in dissipation were significantly different. It is worth noting that upon visualizing cell detachment post-treatment using bright-field images, almost 90 min into the experiment, we observed similar detachment patterns in every cell subtype with much earlier responses recorded in the 50 nM DTX treatment group compared to the 20 nM treatment group (Figure S5).

We combined the 20 nM DTX-QCM-D recordings with actin fluorescence staining (Figure 4c, 4d) and time-lapse confocal imaging (Videos S7–S9) to track biomechanical changes post-treatment at different time points in terms of actin remodeling and cellular rounding. By examining untreated control cells, disparities in the actin organization and cell area of the three subtypes can be visualized immediately and are in line with each cell’s metastatic potential. The low metastatic Cx43D cells displayed long and organized networks of actin filaments spanning almost homogeneously across the cell. As the metastatic potential of the cells increases, actin filaments become less organized and severely fragmented, as observed in the shCx43 cells and, to a lesser extent, in WT cells (Figure 4c, control panel). Upon treatment, cells demonstrated major actin remodeling in terms of dynamics and distribution within treated cells over time. In all cell subtypes, with differences in response speed and strength, DTX caused a major redistribution of the actin filaments toward the cell center away from the cell periphery, eliciting changes in cell shape, notable cell roundness, and a decrease in the cell area, a sign of cell stiffening and detachment.^26,27^ The latter was recorded with a QCM-D, where frequency drops accounting for cell stiffening were observed in all cell subtypes. Interestingly, shCx43 cells experienced the fastest cell area reduction only 5 min into the experiment, losing almost 30% of their total area due to actin stabilization and redistribution, in line with the sharp 60 Hz decrease in its frequency detachment profile (orange curve in Figure 4a, 4d, middle panel). On the contrary, 5 min post-treatment, the parental WT and Cx43D cells barely had any reduction in their cell area. Distinctly, these cells experienced major cell area shrinking (around 50% of total area) followed by clear cell rounding almost 15–30 min into the treatment, at a later stage compared to shCx43 cells. The actin filaments, which were relatively organized compared to those in shCx43, took more time to redistribute into the central part of the cells, as seen in Figure 4c, 4d; thus, the delayed small frequency drops (almost 20 Hz around 40 min into the treatment) as seen in the blue and gray curves in Figure 4a. We then mapped the QCM-D cell detachment responses with time-lapse videos. Although the highly metastatic shCx43 cells showed the fastest response in QCM-D data and actin remodeling, their detachment and morphological changes appeared to be gradual and individualistic, lasting roughly 60 min for the monolayer of cells to begin to round and then detach in a single-cell state. In fact, after 30 min of treatment, shCx43 cells exhibiting a mesenchymal-like phenotype were the most resistant and barely demonstrated any form of cell blebbing (Video S8). However, at a low metastatic state, the Cx43D cell detachment response was unique, where cells in monolayers started to round and form bulging membranes before detaching (Video S9). On the other hand, the control parental WT cells took an intermediate time (30 min) to round and start detaching from the substrate, with responses similar to shCx43 but to a lesser extent and in a slower manner (Video S7). Note that cell viability was not affected by the DTX treatment tested after 90 min, with only 3–5% of cell death reported in all groups.

## Discussion

In this article, we explored the progressive transformation of biomechanical properties of cancer cells across the spectrum of metastatic potential derived from the same cell line by regulating Cx43 expression. Some studies have shown that aggressive cancer cells can potentially adaptively soften, disseminate from their microenvironment, elongate to squeeze through capillaries, and metastasize into distant body sites, forming life-threatening metastatic foci.^28−55^ Cell biomechanics plays a significant role in cancer initiation and progression. Thus, understanding the mechanical fingerprints of cancerous cells with different malignant potentials can provide insights into invasion and metastasis and may improve our ability to physically monitor the formation of circulating tumor cells (CTC) and predict their invasive behavior.

Studies from our laboratory have shown that the overexpression of Cx43 in human TNBC breast cancer cells favors the mesenchymal to epithelial transition (MET), reducing cell proliferation, invasiveness, xenograft tumor onset and growth, metastasis, and restoring cellular differentiation capacity, thus suggesting that Cx43 has tumor suppressive roles. These nonmetastatic cells have a larger cell height, a larger cell surface area, and consequently a larger cell volume. On the other hand, the downregulation of Cx43 has been linked to the downregulation of these epithelial characteristics, including dissolution of cell–cell junctions, adherent junctions, and loss of epithelial polarity, a combination of precursor traits for metastasis. As a result, cells will acquire a mesenchymal phenotype characterized by actin reorganization and stress fiber formation, with the ability of cells to migrate and invade. These cells are smaller in terms of volume, height, and cell surface area.^19,32−34^ Previous efforts showed that metastatic cells have high deformability compared to their nonmetastatic counterparts but often employed atomic force microscopy (AFM)-based SCFS and different cell lines. The interpretation of most of these studies is complicated due to many of the shortcomings associated with AFM, ranging from low precision measurements to the use of coated tips, which interact with the detaching cells, introducing measurement variabilities, in addition to the inability to discern whether the differences arise from the intrinsic genetic properties associated with the culture models adapted or the actual impact of metastatic potential on the biomechanical aspects of the cells. Our work leverages the innovative high throughput and high precision of fluidic-based SCFS of a unique breast cancer *in vitro* progression model of metastasis derived from the same cell line lineage.^29,31,35,36^ This approach enabled us to acquire comprehensive information about the critical mechanical properties of breast cancer cells contingent on the level of their malignant potential. By comparing the adhesion forces of individual cells to cells in clusters, we were able to measure the intercellular adhesion forces of well-established mature tight and gap junctions.^23,37^ Surprisingly, SCFS adhesion data showed that the differences in cellular mechanical properties are masked when MDA-MB-231 cells are not in contact with adjacent cells (*i.e.*, individual cell state), irrespective of Cx43 expression and metastatic potential. However, this mechanical uniformity dissipates when the same cells are adherent to surrounding cells, indicating a complex interplay of cell–cell contacts in dictating cellular mechanical properties and a proportional relationship between cellular adhesion strength and stiffness with the level of cell malignancy when cells are within a cluster. The elevation of *D*_max_ in nonmetastatic cells within a cluster, compared to parental and highly invasive cells, is attributed to the presence of tight and gap junctional proteins and the larger cellular height and volume, thus requiring the retracting cantilever more distance to fully detach the cells. When estimating the apparent cell elasticity, which correlates cell elongation to its adhesion strength, we observed that nonmetastatic cells in clusters have small deformation potential, aligning with a stiffer/rigid phenotype and consistent with their noninvasive state,^38−40^ a distinct feature absent when the cells are not in contact with neighboring cells. Upon inspection of the steepness of the *F*–*D* curves, cells in individual cell states have almost the same slope steepness, unlike cells in clusters. In the latter, the steepest slope was recorded for nonmetastatic cells within a cluster, implying an increase in stiffness, in contrast to the elastic aggressive and highly metastatic cells. In fact, normal/nontumorigenic cells usually form highly developed, polymerized, and well-distributed actin filaments, which contribute to cell rigidity and stiffness in contrast to their tumorigenic counterparts.^7,8^ We confirmed the latter with actin filament staining to study its localization and density in the three cell subsets. While these biomechanical observations related to Cx43-modulated cells are novel, the association of cells’ mechanical properties and their use as biomarkers to determine the malignant potential of cells has been reported in other metastatic cell models.^8−10,31,55,41^

We complemented the fluidic-based SCFS data with QCM-D measurements where frequency shifts indicate differences in the convoluted viscoelastic behavior of the cell membrane and the underlying ECM and substrate (the layer providing direct mechanical coupling between the matrix/sensor surface and the cell membrane^42^), and dissipation shifts offer insights into the viscoelasticity and rigidity of the cell membrane of detaching cells.^43^ It is worth noting that cells represent an acoustically thick layer (between 10 and 20 μm) much larger than the characteristic penetration depth (δ) (>250 nm in water at *f*_0_ = 5 MHz), thus the layer just above the sensor (*i.e.*, cell membrane, cytoskeleton, etc.) influence QCM-D measurements. Our observations showed a typical Δ*F* and Δ*D* behavior upon cell detachment when trypsin was introduced.^44−46^ An increase in Δ*F* indicated that a lesser mass was detected at the sensor’s surface, confirming cell detachment and cytoskeletal disruption. In fact, the magnitude of the frequency shifts was directly proportional to cell density and varied significantly between the three subtypes at high cell density only when tight cell–cell contacts existed between the cells. The same Δ*F*-response trend was observed in low cell count but to a lesser extent, which could be attributed to a lesser mass of cells cultured on top of the sensor’s area. Our data revealed a strong correlation between Δ*D*-responses and cell malignancy levels, influenced by the cellular mechanical properties, cytoskeletal rearrangements, and the number of focal adhesion points but not cell–cell contacts. Highly invasive cells dissipated more energy in both cell confluencies, indicating a viscoelastic cell membrane, an intrinsic cell property. In addition, when comparing Δ*D* shift magnitudes, we observed that nonmalignant cells in both cell densities had a smaller response, confirming a decrease in the viscous character and higher rigidity of the cell membrane, contrary to the metastatic cells. In addition, a higher *Df*-slope suggests that the cell layer is more viscoelastic (softer and more dissipative) and has a less rigid phenotype. Our data showed that on the spectrum of metastatic potential, noninvasive cells have a viscous cell membrane and a stiff cytoskeleton with elevated rigidity, whereas metastatic cells exhibited a lesser viscous membrane but a softer cytoskeleton consistent with the existing literature.^10,47,48^ Confocal fluorescence images confirmed that low metastatic cells form relatively well-organized filamentous actin structures with longer fibers distributed homogeneously from the edge to the cell center, explaining the stiff phenotype. In contrast, highly metastatic cells had a more elastic and softer phenotype possibly due to actin distribution at the edge of the cell rather than its center. Shifts in dissipation and *Df*-plots are far more sensitive measurements to assess for membrane viscosity and elasticity than frequency shifts. This is reflected by the level of dissipation events occurring during cellular detachment, such as the viscous slip between the cell basal membrane and the liquid medium trapped between the cells and the sensor surface, the remodeling of actin stress fibers, and the frictional slip between integrins and the ECM.^45^

Finally, we used QCM-D coupled with fluorescence staining to determine the biomechanical cellular responses to DTX treatment at different concentrations and time points on the three cell subtypes with a spectrum of the metastatic state. DTX treatments unveiled time-dependent alterations in the mechanical properties of the basal region of the treated cells. Upon DTX injection, metastatic cells on the higher end of the metastatic potential range exhibited an immediate and profound response in Δ*F* and Δ*D*, nearly 6-fold higher than cells on the lower end of the metastatic state. Even though treated cells tend to round up and lift off, there was a marked negative shift in frequency with a positive shift in dissipation, after which both signals reverted to the baseline. This sequence of responses suggests that cells initially become rigid and stiff due to microtubule stabilization and then round up before detachment. The same pattern of responses was observed in the other two cell subtypes but to a lesser extent and at a much slower pace. This differential response might be due to the inherent disorganized and altered cytoskeleton of highly metastatic cells, rendering them prone to DTX treatment and sensitive to changes in the microtubule dynamics compared with the less invasive counterparts. In addition, sequential imaging of treated cells revealed that highly metastatic cells predominantly exhibit bulk detachment of cells (in groups), whereas the counterparts typically detach individually (Figure S5). Our observations are consistent with the reported effects of DTX on cells.^27,49,50^ Although DTX primarily affects microtubules, it can also have secondary effects on other cytoskeletal components within the cell, mainly the redistribution of actin filaments away from the cell periphery toward the cell center,^27,49,51^ which was observed by fluorescence staining. The induced effects on actin dynamics result in the formation of thicker, more stable stress fibers, resulting in increased cellular rigidity. Biologically, such treatments could render cancer cells less deformable, potentially impeding their ability to invade and metastasize to other tissues.

Our analysis provides coherent evidence of the unfolding biomechanical changes of breast epithelial cancer cells when their malignant potential varies across the different states of EMT and MET. As the cell’s malignant potential goes up from the noninvasive Cx43D cells to the aggressive WT cells and the highly invasive shCx43 cells in the spectrum of metastasis, cells become less adhesive and more viscoelastic and deformable. The larger the cell deformability and viscoelasticity are, the greater the contact surface between the detached soft cells and the surrounding endothelial cells, giving the cells more opportunities to form adhesion bonds and favoring tumor initiation.^52^

## Conclusions

Our work represents a stepping stone to understanding how mechanical changes in cancer cells may determine how cancer progresses and responds to chemotherapeutic agents. It is important to note some potential areas for expansion as we only used one cell line, which could be extended to include additional breast cancer cell lines of different breast cancer types, thereby enriching our understanding of the various cancer behaviors and responses. In addition, while our work holds promise for applications in label-free CTC detection and sensing platforms, challenges remain in this field, particularly the genetic and biomechanical heterogeneity of CTCs. The role of tumor cell biomechanics during the different stages of metastasis and chemotherapeutic treatments is overlooked and is more intricate than it seems. By comprehending our data, we can question the overly simplistic notion that metastatic tumors are uniformly soft. In reality, their mechanical properties, including both softness and stiffness, fluctuate depending on various invasive stages.

## Materials and Methods

### Cell Culture

MDA-MB-231 human epithelial breast cancer cells were routinely passaged in Dulbecco’s modified Eagle’s medium (DMEM)—high glucose (Sigma, D5796, St. Louis, MO) supplemented with 10% fetal bovine serum (FBS) (Sigma, F-9665) and 1% penicillin–streptomycin (Lonza, Basel, Switzerland, DE16-602E) at 37 °C and 5% CO_2_ in a humidified incubator. MDA-MB-231 cells (WT) are regarded as triple-negative metastatic human mammary adenocarcinoma cells. To generate cells with different metastatic potentials by modulating Cx43 expression, MDA-MB-231 cells with downregulated and upregulated Cx43 expression were generated as previously described in ref (19).

Briefly, MDA-MB-231 with overexpression of Cx43 (Cx43D) in fusion with Dendra-2, a photoconvertible fluorescent protein, were generated by transduction with pCSCW-Dendra-2-Cx43 lentiviral particles and sorted by FACS Aria III SORP (BD Biosciences, San Jose, CA) according to their fluorescence intensity. Cx43D cells express a fusion protein of Dendra-2 fused at the N-terminus of Cx43 and can form functional gap junctions. On the other hand, MDA-MB-231 cells with downregulation of Cx43 expression (shCx43) were transfected with a pGFP-V-RS plasmid vector expressing GFP with the puromycin resistance gene and an inserted shRNA (one of four different constructs) directed against Cx43 (OriGene Technologies, cat#: TR30007, Rockville, MD) using the Lipofectamine 2000 reagent (Invitrogen, Carlsbad, CA) according to the manufacturer’s instructions. The transfected cells were then enriched by FACS sorting in a FACS Aria III SORP, routinely selected in culture using 0.5 μg/mL puromycin and maintained in 0.1 μg puromycin.

### High-Throughput Single-Cell Adhesion Force Spectroscopy Using FluidFM

Single-cell force spectroscopy of single cells individually or in cluster states was performed using a FluidFM (OMNIUM system) instrument (Cytosurge AG., Zurich, Switzerland) placed on a vibration-free table. Rectangular FluidFM silicone micropipette cantilevers with an aperture diameter of 8 μm and a nominal spring constant of 2 N/m were mounted on the *z*-stage of the OMNIUM system (termed head) and used to perform cellular detachment experiments. Before SCFS measurement, the cantilever spring constant (*k* [N/m]) was calibrated in air using the Sader method by a built-in function in the OMNIUM system.^53^ Accordingly, the inverse optical lever sensitivity (*InvOLS* or β [μm/V]) was defined based on the position of the laser reflection optics of the instrument, and its accuracy directly influences the measurements of the force values as previously described in ref (54). The bending of the cantilever was measured through an incident optical beam on the surface of the probe, and the deflected beam was captured by a photodetector in the form of an electrical signal measured in volts (V). The force of adhesion [*F*_adh_ (N)] is directly proportional to the spring constant (*k*), the deflection sensitivity (β), and the measured deflection (*V*), and it fulfills Hook’s law: *F* = *k* × *x* = *k* × β × *V*, where *x* is the deflection of the cantilever [m] after multiplying the deflection signal by the deflection sensitivity.^3^ The SCFS measurement process yields the characteristic force–distance (*F*–*D* curve) where the maximum peak force recorded during the cantilever deflection corresponds to the maximum force required to fully detach a cell from its underlying substrate and represents the adhesion force of a cell (*F*_adh_). On the other hand, the maximum detachment distance (*D*_max_ [μm]) represents the distance from the surface at which the cell exerts maximal force; it is also the distance traveled by the cantilever to fully detach the cell from the substrate.

MDA-MB-231 WT, shCx43, and Cx43D were seeded separately in 6-well tissue culture plastic plates (Corning) at densities of 7.5 × 10^4^ cells/mL for ease of cell selection in a single-cell state or in a cell within a cluster state. After 24 h of cell seeding, the six-well plate was loaded onto the OMNIUM system incorporated within a humidified incubator kept at 37 °C during the experiment. Preceding the adhesion force measurements, the hollow micropipette cantilever was filled with 1 μL of glycerol and Milli-Q water at a 1:1 (v/v) ratio. When performing the SCFS measurements, the cantilever is set to approach the selected cell at a speed of 1 μm/s, then pausing for 5 s when it intercepts with the targeted cell, which is detected when the deflection of the photodetector voltage reaches 25 mV. Herein, the cantilever remains immobile for 5 s while applying a negative pressure ranging between −600 and −800 mbar. The cantilever then retracts from the surface, bending downward due to the adhesive force between the cell, the substrate, and the surrounding cells, if any. When the force required to bend the cantilever exceeds the maximum cellular adhesion force, the cell detaches from its substrate with multiple rupture events and the cantilever returns to its original state. The cantilever retraction distance post cell detachment is preserved at 50 μm for all experiments. After each adhesion force measurement, a cleaning process was performed to prevent cell debris from accumulating at the cantilever tip and ensure accurate subsequent measurements. The cleaning process begins by rinsing the cantilever with 10% terga-zyme (source) for 3 min, washing it three times with Milli-Q water, and finally rinsing it with cell culture media. Including the cleaning cycles, a maximum of 6–8 cells can be detached per hour, and between 20–30 cells from different cell batches were analyzed per cell subtype.

The selection criteria for the cells of interest were determined by the number of surrounding cells: for individual cells, the cell of interest had to be clear of any neighboring cells (Figure S3, upper panel). As for cells in clusters, the cell of interest must be adherent to at least two neighboring cells (Figure S3, lower panel). The relative force change was calculated according to the following equation
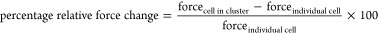


### Docetaxel Chemosensitivity and Trypan Blue Exclusion Assay

Docetaxel (DTX) (MedChemExpress (MCE), HY-B0011, NJ) was prepared in stock solutions of 1 μM in dimethyl sulfoxide (DMSO). Cells were seeded in 12-well tissue culture plates at 10^5^ cells/mL cell density and treated with DTX (0, 20, and 50 nM concentrations) 24 h post seeding. The effect of DTX on cellular morphology and adhesion was observed using an inverted microscope by imaging the cells at preset time intervals (0, 30, 60, 90, 120 min). After the final imaging interval, cells from different conditions were collected by trypsinization and counted using the trypan blue exclusion assay using a hemocytometer.

### Quartz Crystal Microbalance with Dissipation Monitoring

QCM-D experiments were performed using a Q-Sense E4 device (BiolinScientific/Q-Sense E4, Gothenburg, Sweden) equipped with optically polished gold-coated quartz sensors (QSX 301) with a fundamental frequency (*f*_0_) of 5 Hz to record variations in resonant frequency (Δ*F*) and energy dissipation (Δ*D*) as a function of time at the order of overtone *n* = 3. Prior to experiments, the sensors were cleaned using a UV-ozone cleaner for 10 min, followed by dipping the sensors in a mixture of Milli-Q water, hydrogen peroxide, and ammonia (volume ratio of 5:1:1) heated to 75 °C for 5 min. The sensors were then rinsed thoroughly with Milli-Q water and dried with a gentle stream of nitrogen gas. Later, the sensors were dipped in 70% ethanol solution for 20 min and rinsed with 1× PBS, followed by media to prepare their surfaces for cell seeding. Post cell seeding and at the set time point, the sensors were rinsed with 1× PBS, their back was dried gently with a Kimwipe to remove residual liquid, and then mounted in a flow module (Q-sense) set at 37 °C. Once mounted on the QCM-D module, the fundamental frequency of each sensor was detected to create a baseline for frequency and dissipation measurements at the third, fifth, and seventh harmonics. Prewarmed DMEM high glucose cell culture media was injected for at least 1 h using a peristaltic pump at 20 μL/min until all of the harmonics of interest had stabilized. After stable baselines were achieved, the flow and buffers injected were adjusted according to the desired experimental protocol, and Δ*F* and Δ*D* were recorded simultaneously.

When QCM-D measurements are performed in the gas phase, the shift in frequency is proportional to the layer of mass adhered onto the sensor surface, such that it obeys the Sauerbrey model
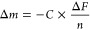
where Δ*m* corresponds to the change in the mass-adhered material, and *C* represents the mass sensitivity constant of the sensor. However, the Sauerbrey model is only applicable when there is no change in dissipation and is suitable for uniform and rigid materials even when measurements are performed in liquid phases. Since this estimation is independent of the viscoelastic properties of the adhered layer, the Sauerbrey model must be corrected to include the shear elastic (storage modulus *G*_s_) and viscosity (loss modulus *G*_L_) components of the complex shear modulus G* such that
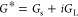
The corrected relation is termed the Voigt model, and its elements assume that the changes in frequency and dissipation are dependent on the viscosity and complex shear modulus of the studied material. Similarly, the change in dissipation obeys the Voigt model and is dependent on the material adhered to the sensor surface and the measurement phase (air/liquid) such that
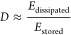


### Viscoelasticity Assay

On clean and ethanol-sterilized QCM gold sensors, MDA-MB-231 WT, shCx43, and Cx43D on the surface of the sensors were at low (6.5 × 10^4^ cells/sensor) and high (2 × 10^5^ cells/sensor) cellular densities to examine the role of connexin expression (which also reflects the invasive phenotype of cells) in the mechanical properties of the breast cancer cells. The cells were dripped gently on top of the sensor area for 20 min, after which complete media was added to the Petri dish. The cells were then incubated at 37 °C for 24 h before the QCM-D experiment to ensure proper cell–substrate adhesion. After mounting the sensors on QCM-D flow modules and stabilizing the baseline, 1× PBS was injected for 10 min to remove any traces of FBS, followed by trypsin with ethylenediaminetetraacetic acid (EDTA) injection (30 μL/min for 35 min) to allow for cell detachment. Finally, 1× PBS was injected again with a high flow rate for 10 min to remove unbound cells off the sensors’ surface. The detachment response of cells to trypsin was characterized by the maximal frequency shifts, maximal dissipation shifts, and variation of dissipation with respect to frequency.

### DTX Treatment Assay

Cells were seeded on the surfaces of the sensors for 24 h prior to the experiment. The sensors were then mounted on the QCM-D modules, and the stabilization protocol was conducted. Prewarmed media was injected for 10 min, and then the cells were treated with DTX at different doses (0, 20, 50 nM) for 2 h at a very slow flow rate (6 μL/min), after which 1× PBS was injected at a high flow rate for 30 min to remove detached cells off the surface of the sensors. As a negative control, cells were exposed to a continuous flow of DTX or medium to rule out the impact of shear flow on the recorded frequency and dissipation responses.

### Holographic Imaging of Cells

Cells were seeded at 75,000 cells (surface area) on top of gold-sputtered coverslips for reflective surface purposes. A digital holographic microscope (DHM, Lyncée Tec, Switzerland) with holographic interferometry technology was used at a 10× objective to characterize cross-sectional cell heights of MDA-MB-231 cells with varying Cx43 expression. A cross-sectional profile line is drawn midway through the adherent cell to ensure that the entire curvature of the cell is accounted for in the height measurement. The underlying substrate on which the cells were adhered to is assigned as the zero-height reference point, *i.e.*, the baseline. The total height of the cell is then measured, encompassing the entire cell profile from the baseline to the baseline at both ends of the cell, including the highest point of the cell height. The measurements of cell height were calculated and calibrated using 10 μm polystyrene beads (01-00-104, micromod, Partikeltechnologie, GmbH).

### Fluorescence and Time-Lapse Imaging

Cells were cultured on sterile glass coverslips for 24 h and treated with 20 nM DTX. The treatment was stopped at different time points, where the cells were fixed for 15 min with 4% paraformaldehyde, permeabilized with 0.1% Triton-X for 15 min, and then incubated with phalloidin-TRIC (2 μg/mL) for 1 h to stain for actin. The nuclei of the cells were counterstained with DAPI. The coverslips were then mounted on glass microscopic slides by using Prolong Antifade (Molecular Probes). The cells were examined and imaged with a laser-scanning confocal microscope (Zeiss, LSM710). For time-lapse experiments, cells were seeded onto glass-bottom culture dishes (confocal dishes) for 24 h for real-time live confocal fluorescence microscopy. Bright-field and fluorescence images were taken simultaneously to monitor transfected cells with Dendra and GFP proteins being excited with the 488 nm line. Pretreatment images of cells were taken for five iterations at a 20-min interval, after which the cells were treated with 20 nM DTX and imaged every 2 min for a total period of 60 min.

### Data Evaluation and Statistical Analysis

Analysis of the obtained SCFS data was carried out in a custom MATLAB code to evaluate the characteristic *F*–*D* curves and calculate the *F*_adh_, and *D*_max_. Lognormal and normal distribution curve fitting, data plots, and significance tests were carried out in GraphPad Prism 8.0.1 and OriginPro 9.5. For the raw data, outliers were removed using the ROUT method with a significance level of 0.05 and accordingly discarded. For QCM-D, data analysis was performed using QTools software and NBS-QCManalysis software. Statistical evaluation was carried out by analysis of variance (ANOVA) followed by post hoc Tukey’s multiple comparison test. Results are presented as means ± SEM from at least three independent replicates. Significance levels were assigned by * for *p* < 0.05, ** for *p* < 0.01, and *** for *p* < 0.001. Independent cell cultures usually refer to different passages and batches.

## Data Availability

All data produced in this study and used for analysis and drawing conclusions are either present in the paper and/or the Supporting Information. Additional data needed may be requested from the authors.

## Supporting Information Available

qRT-PCR validation of Cx43 expression; measurements of cell diameter and surface area; SCFS schematic; normalized QCM-D frequency and dissipation data to cell count; captions of supplementary videos of FluidFM-based SCFS detachment of cells; and captions for supplementary videos depicting real-time responses of DTX treated cells (PDF)
Detachment of an individual WT cell using FluidFM-based SCFS (Video S1) (MP4)
Detachment of an individual shCx43 cell using FluidFM-based SCFS (Video S2) (MP4)
Detachment of an individual Cx43D cell using FluidFM-based SCFS (Video S3) (MP4)
Detachment of a WT cell in a cluster using FluidFM-based SCFS (Video S4) (MP4)
Detachment of a shCx43 cell in a cluster using FluidFM-based SCFS (Video S5) (MP4)
Detachment of a Cx43D cell in a cluster using FluidFM-based SCFS (Video S6) (MP4)
Real-time response of WT cells treated with 20nM DTX for 60 min (Video S7) (AVI)
Real-time response of shCx43 cells treated with 20nM DTX for 60 min (Video S8) (AVI)
Real-time response of Cx43D cells treated with 20nM DTX for 60 min (Video S9) (AVI)

## Supplementary Material

am4c06335_si_007.mp4

am4c06335_si_008.avi

am4c06335_si_009.avi

am4c06335_si_010.avi
