# Supplementary material for: Single-Cell Fluidic Force Spectroscopy Reveals Dynamic Mechanical Fingerprints of Malignancy in Breast Cancer
Source: ACS Appl Mater Interfaces. 2024 Aug 6;16(38):50147–59. doi: 10.1021/acsami.4c06335 (PMC11440459; doi:10.1021/acsami.4c06335)
Supplement: Supplementary file 1 — am4c06335_si_001.pdf [file am4c06335_si_001.pdf]

Supporting Information for

Single-cell Fluidic Force Spectroscopy Reveals Dynamic Mechanical Fingerprints of Malignancy in Breast Cancer

Zeina Habli[a], *Ahmad Zantout*[a], *Nadine Al-Haj*[b], *Raya Saab*[c], *Marwan El-Sabban*[b*], *Massoud L. Khraiche*[a*]

[a] Neural Engineering and Nanobiosensors Group, Biomedical Engineering Program, Maroun Semaan Faculty of Engineering and Architecture, American University of Beirut, Beirut 1107 2020, Lebanon.
[b] Department of Anatomy, Cell Biology, and Physiological Sciences, Faculty of Medicine, American University of Beirut, Beirut 1107 2020, Lebanon.
[c] Department of Pediatrics, Stanford University School of Medicine, Palo Alto, CA 94304, USA.

[*] Corresponding authors

Corresponding Authors emails:
mkhraiche@mail.aub.edu and me00@aub.edu.lb

This PDF file includes:

Figures: S1 to S5
Table S1
Movies S1 to S9

Validation of Cx43 expression was done routinely in sorted shCx43 and Cx43D cells using qRT-PCR. Total RNA was extracted using NucleoSpin RNA II Kit (Macherey-Nagel, Düren, Germany). Single-stranded cDNA synthesis was performed RevertAid first strand cDNA synthesis kit (ThermoFisher, Vilnius, Lithuania) with a total of 1µg of according to manufacturer instructions. Real-time quantitative polymerase chain reaction (RT-qPCR) was carried out using iQ SYBR Green Supermix in a CFX96 system (Bio-Rad Laboratories, Hercules, CA, USA) with the needed primers listed in table S1. The ΔΔCq method was applied to calculate the relative fold change in gene expression after normalization to GAPDH first then to Cx43 expression in the WT cells as depicted in figure S1.

Gene	Primer Sequence	Annealing Temperature (°C)
hCx43	5’-CTTCACTACTTTTAAGCAAAAGAG-3’<br>3’-TCCCTCCAGCAGTTGAG-5’	52
hGAPDH	5’-TGGTGCTCAGTGTAGCCCAG-3’<br>3’-GGACCTGACCTGCCGTCTAG-5’	58

Table S1: List of primers for qRT-PCR. Cx43: Connexin 43, GAPDH: Glyceraldehyde 3-phosphate dehydrogenase, h: human.

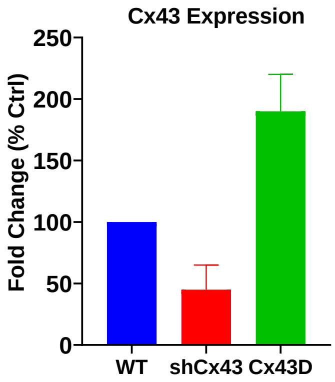

Figure S1: Down-regulation or up-regulation of Cx43 in MDA-MB-231 cells. Bar graph representing Cx43 mRNA expression in MDA-MB-231 WT, shCx43, and Cx43D cells as detected by qPCR and normalized to Cx43 expression in WT cells (control). The values depicted are the mean ±SEM from at least three separate experiments.

Using ImageJ, cell surface area was measured using bright field images of confluent cells (n>30 per cell subtype). Significant differences in cell area among the three subtypes was observed. The highly metastatic shCx43 cells exhibited the smallest cell surface area, while the WT cells had an intermediate cell area, and the non-metastatic Cx43D cells showed the largest cell area (Figure S2a). As for cell diameter, minimal differences were observed of cells in suspension after trypsinization suggesting that the diameter is relatively consistent when cells are not adhered to a substrate (Figure S2b).

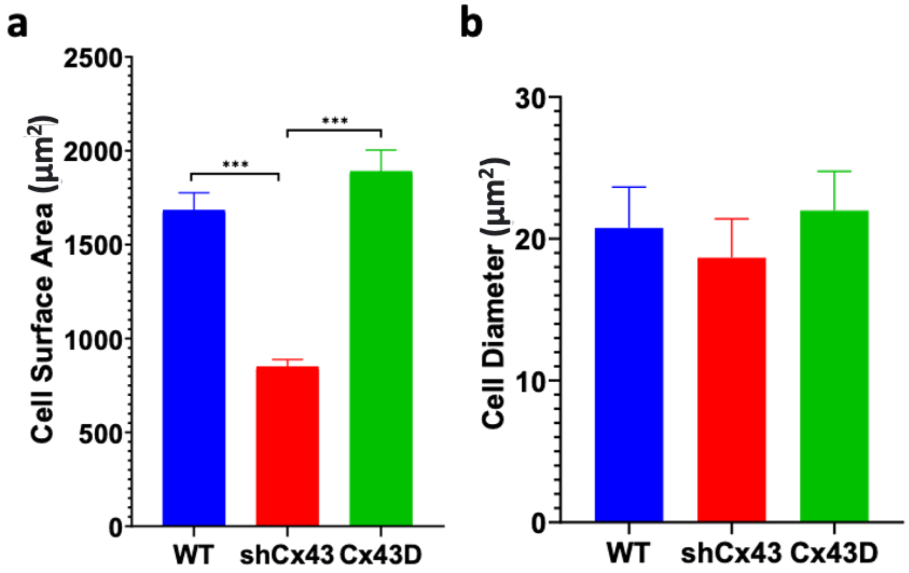

Figure S2. Measurements of cell surface area and cell diameter of MDA-MB-231 WT, shCx43, and Cx43D cells using imageJ. a) Average cell surface area and **b)** average diameter from at least 30 single cells in each Cx43 expression condition. The values depicted are the mean ±SEM from three separate experiments. *** denotes a p-value < 0.001, ** denotes a p-value < 0.01, and * denotes a p-value < 0.05 compared to different conditions using ANOVA followed by Post-hoc Tukey HSD test.

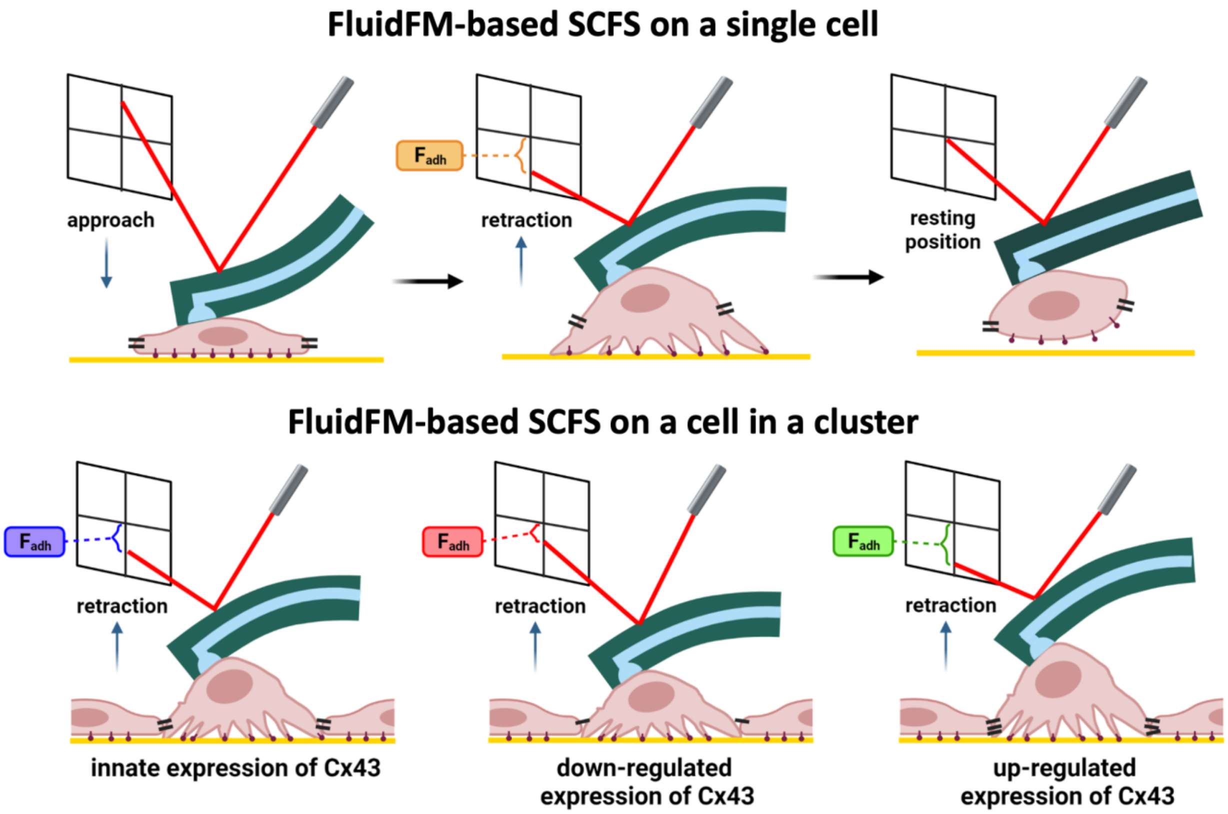

Figure S3. Schematic showing FluidFM-based single-cell force spectroscopy on a single cell not adhering to neighboring cells (upper panel) and on a single cell in a cluster (lower panel) where the cell of interest is adherent to at least two neighboring cells.

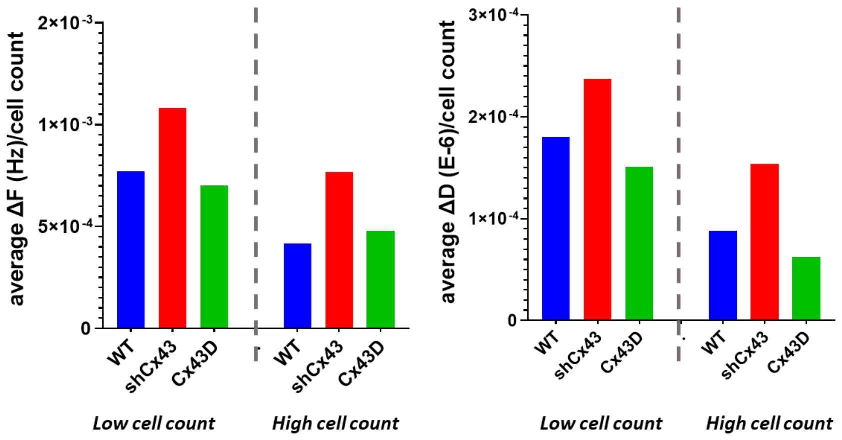

Figure S4. Metastatic state of MDA-MB-231 cells influences cellular viscoelasticity revealed by QCM-D data normalized to cell density. Changes in ΔF- and ΔD- responses normalized to cell count of MDA-MB-231 cells with varying expression of Cx43 undergoing cell detachment.

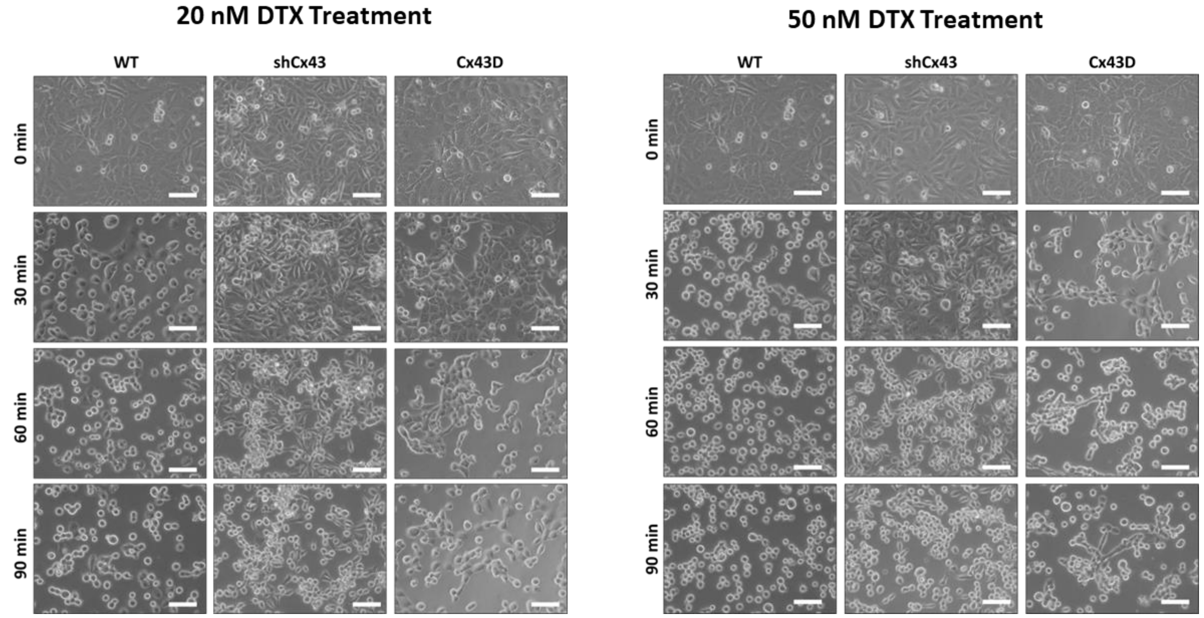

Figure S5: Metastatic state of MDA-MB-231 cells affects cell responses, in real-time, to DTX treatments. Bright filed images of the treated three cell-subsets at different time points and DTX concentrations. Scale bar is 50 μm.

Videos caption:

Video S1: Detachment of an individual WT cell using FluidFM-based SCFS

Video S2: Detachment of an individual shCx43 cell using FluidFM-based SCFS

Video S3: Detachment of an individual Cx43D cell using FluidFM-based SCFS

Video S4: Detachment of a WT cell in a cluster using FluidFM-based SCFS

Video S5: Detachment of a shCx43 cell in a cluster using FluidFM-based SCFS

Video S6: Detachment of a Cx43D cell in a cluster using FluidFM-based SCFS

Video S7: Real-time response of WT cells treated with 20nM DTX for 60 min

Video S8: Real-time response of shCx43 cells treated with 20nM DTX for 60 min

Video S9: Real-time response of Cx43D cells treated with 20nM DTX for 60 min
